# Dietary diversity score and incident peptic ulcer in Chinese older adults: a prospective cohort study

**DOI:** 10.3389/fnut.2026.1984205

**Published:** 2026-10-26

**Authors:** Kai Wang, Xiaodan Li

**Affiliations:** 1 Department of Colorectal Surgery, Heping Hospital Affiliated to Changzhi Medical College, Changzhi, Shanxi, China; 2 Department of Pediatric Health Care, Zhangzi County Maternal and Child Health Family Planning Service Center, Changzhi, Shanxi, China

**Keywords:** Chinese longitudinal healthy longevity survey (CLHLS), dietary diversity score (DDS), older adults, peptic ulcer, prospective cohort study

## Abstract

**Objective:**

To examine the association between dietary diversity score (DDS) and incident peptic ulcer among older Chinese adults.

**Methods:**

We analyzed four waves of CLHLS data (2008–2018) from 13,274 ulcer-free adults aged ≥65 years with complete covariate data. DDS (0–9) was derived from a 9-item food frequency questionnaire. Cox proportional hazards models and restricted cubic splines were used to estimate hazard ratios, adjusting for sociodemographic, lifestyle, and health-related covariates. Sensitivity analyses included competing-risk models, lagged exposure updates, hospital-confirmed diagnoses, and multiple imputation.

**Results:**

In fully adjusted models, each 1-point increase in DDS was associated with a lower risk of incident ulcer (HR = 0.944, 95% CI 0.904–0.986; *p* = 0.009). Compared with the lowest tertile, the highest tertile had a 24% lower risk (HR = 0.757, 95% CI 0.618–0.927; *p* = 0.007), with an approximately linear dose–response relationship (*p* for nonlinearity = 0.655). The association was consistent in most subgroups but was absent among participants with disability in activities of daily living (*p* for interaction = 0.004). Findings were robust across sensitivity analyses, although the association attenuated to null after 3 years of follow-up and in the 6-year landmark analysis, suggesting that the effect was mainly evident during early follow-up.

**Conclusion:**

Higher dietary diversity was associated with a lower risk of incident peptic ulcer among older Chinese adults, particularly during early follow-up and among functionally independent individuals. Randomized trials are needed to confirm causality and evaluate the intervention potential of dietary diversity.

## 1 Introduction

Peptic ulcer disease is a chronic mucosal defect of the stomach or duodenum, with a global lifetime prevalence of approximately 5–10%, and is the most common cause of upper gastrointestinal bleeding and perforation (1). In China, the prevalence of peptic ulcer disease among adults is also relatively high (2). With increasing age, the incidence of ulcer-related bleeding and perforation, the risk of rebleeding, and case-fatality rates all increase substantially. In addition, older adults often face a higher risk of adverse outcomes because of multiple comorbidities and the frequent use of non-steroidal anti-inflammatory drugs (NSAIDs), making peptic ulcer disease an important gastrointestinal disease burden in aging societies (3). *Helicobacter pylori* (*H. pylori*) infection and NSAIDs use are the two major causes of peptic ulcer disease. In China, the prevalence of *H. pylori* infection remained high during 2014–2023 (4), and NSAIDs-related ulcers are particularly common in older adults (5). In addition to these two major causes, dietary factors are considered modifiable risk factors; however, evidence on their association with the development of peptic ulcer disease in older adults remains limited.

The dietary diversity score (DDS) is an indicator used to assess individual nutritional adequacy. By summing the number of food groups consumed daily, it reflects overall diet quality and is widely used as an exposure measure in nutritional epidemiology (6). A diversified diet helps ensure adequate intake of protein, vitamins, and trace elements, maintains the integrity of the gastrointestinal mucosal barrier, and contributes to anti-inflammatory and antioxidant states (7), thereby potentially influencing ulcer occurrence. Previous studies have reported that dietary fiber intake is inversely associated with the risk of duodenal ulcer among men in the United States (8). Kurata et al. also found a close association between dietary factors and ulcer risk (9). However, these studies focused on single nutrients or specific dietary patterns, and evidence regarding dietary diversity remains limited.

In the Chinese Longitudinal Healthy Longevity Survey (CLHLS) cohort, DDS has been reported to be associated with multiple health outcomes, including all-cause mortality (6), frailty (10), sarcopenia (11), and disability in activities of daily living (12); however, no study has examined its association with peptic ulcer disease. Because very old adults face a higher risk of ulcer bleeding and perforation, as well as stronger competing risks of death, and because dietary diversity is a modifiable lifestyle intervention target, this study used longitudinal CLHLS data from 2008 to 2018 to investigate the association between dietary diversity and incident peptic ulcer, with the aim of providing dietary guidance for primary prevention of peptic ulcer disease in older adults.

## 2 Methods

### 2.1 Data sources and study population

This prospective cohort study used data from four waves of the CLHLS conducted in 2008, 2011, 2014, and 2018. Using the 2008 survey as the baseline, 16,954 participants were initially included. We sequentially excluded individuals aged <65 years (*n* = 391), those with prevalent peptic ulcer disease at baseline (*n* = 1,132), and those with missing data for key covariates (*n* = 2,157). The final analytic sample comprised 13,274 participants aged ≥65 years who had no history of peptic ulcer disease at baseline and complete data for the covariates required in the main analysis, corresponding to 78.3% of the baseline respondents. Among them, 13,139 participants with complete BMI data were included in the baseline characteristic description. BMI completeness was not part of the primary inclusion criteria: the main analytic sample required complete data only for the covariates in the fully adjusted model. Because Table 1 reports the distribution of BMI, the subgroup analyses stratify by BMI, and sensitivity analysis (E) additionally adjusts for BMI, these analyses required complete BMI data and were therefore conducted in a BMI-complete subsample (*n* = 13,139). BMI was missing for 135 participants (1.0%) of the main sample, and the two samples yielded consistent results. The median age of the sample was 87 years (interquartile range [IQR], 77–94). Missing data were concentrated in the cognitive score (1,849 participants, 11.98%), followed by hypertension (188, 1.22%) and diabetes (121, 0.78%); all other variables had fewer than 45 missing values. The CLHLS was approved by the Ethics Committee of Peking University (approval no. IRB00001052-13074), and written informed consent was obtained from all participants or their family members before each wave of the survey. This study used publicly available, de-identified secondary data and therefore did not require additional ethics approval. All analyses were conducted in accordance with the ethical principles of the Declaration of Helsinki.

**Table 1:** Baseline characteristics of participants by tertiles of dietary diversity score (BMI-complete subsample, *n* = 13,139), CLHLS 2008–2018.

Variable	Total (*n* = 13,139)	T1 (low, *n* = 4,257)	T2 (middle, *n* = 4,400)	T3 (high, *n* = 4,482)	*p* value
Sociodemographic characteristics					
Age, years, median [IQR]	87.0 [77.0, 94.0]	88.0 [79.0, 94.0]	88.0 [78.0, 95.0]	86.0 [75.0, 94.0]	<0.001
Sex, *n* (%)					<0.001
Male	5,891 (44.8)	1746 (41.0)	1911 (43.4)	2,234 (49.8)	
Female	7,248 (55.2)	2,511 (59.0)	2,489 (56.6)	2,248 (50.2)	
Education, *n* (%)					<0.001
Illiterate	7,947 (60.5)	2,970 (69.8)	2,734 (62.1)	2,243 (50.0)	
Primary school	3,832 (29.2)	1,070 (25.1)	1,293 (29.4)	1,469 (32.8)	
Middle school	724 (5.5)	145 (3.4)	214 (4.9)	365 (8.1)	
High school or above	636 (4.8)	72 (1.7)	159 (3.6)	405 (9.0)	
Residence, *n* (%)					<0.001
Urban	5,335 (40.6)	1,141 (26.8)	1810 (41.1)	2,384 (53.2)	
Rural	7,804 (59.4)	3,116 (73.2)	2,590 (58.9)	2098 (46.8)	
Marital status, *n* (%)					<0.001
Married	4,266 (32.5)	1,193 (28.0)	1,397 (31.8)	1,676 (37.4)	
Others	8,873 (67.5)	3,064 (72.0)	3,003 (68.2)	2,806 (62.6)	
Self-rated economic status, *n* (%)					<0.001
Very rich	145 (1.1)	21 (0.5)	45 (1.0)	79 (1.8)	
Rich	1,675 (12.7)	276 (6.5)	515 (11.7)	884 (19.7)	
So so	9,131 (69.5)	2,734 (64.2)	3,224 (73.3)	3,173 (70.8)	
Poor	1839 (14.0)	985 (23.1)	540 (12.3)	314 (7.0)	
Very poor	349 (2.7)	241 (5.7)	76 (1.7)	32 (0.7)	
Lifestyle factors					
Smoking status, *n* (%)					<0.001
Never	8,654 (65.9)	2,956 (69.4)	2,939 (66.8)	2,759 (61.6)	
Former	2,105 (16.0)	596 (14.0)	669 (15.2)	840 (18.7)	
Current	2,380 (18.1)	705 (16.6)	792 (18.0)	883 (19.7)	
Drinking status, *n* (%)					<0.001
Never	9,030 (68.7)	3,070 (72.1)	3,074 (69.9)	2,886 (64.4)	
Former	1786 (13.6)	550 (12.9)	582 (13.2)	654 (14.6)	
Current	2,323 (17.7)	637 (15.0)	744 (16.9)	942 (21.0)	
BMI, kg/m2, median [IQR]	20.0 [17.9, 22.5]	19.5 [17.6, 21.8]	20.0 [17.9, 22.3]	20.5 [18.4, 23.0]	<0.001
BMI category, *n* (%)					<0.001
Underweight	4,133 (31.5)	1,573 (37.0)	1,378 (31.3)	1,182 (26.4)	
Normal	7,144 (54.4)	2,215 (52.0)	2,423 (55.1)	2,506 (55.9)	
Overweight	1,505 (11.5)	382 (9.0)	488 (11.1)	635 (14.2)	
Obese	357 (2.7)	87 (2.0)	111 (2.5)	159 (3.5)	
Chronic conditions					
Hypertension, *n* (%)					0.001
No	10,510 (80.0)	3,483 (81.8)	3,483 (79.2)	3,544 (79.1)	
Yes	2,629 (20.0)	774 (18.2)	917 (20.8)	938 (20.9)	
Diabetes, *n* (%)					<0.001
No	12,790 (97.3)	4,191 (98.4)	4,288 (97.5)	4,311 (96.2)	
Yes	349 (2.7)	66 (1.6)	112 (2.5)	171 (3.8)	
Functional status and cognition					
ADL disability, *n* (%)					0.054
Independent	10,958 (83.4)	3,585 (84.2)	3,682 (83.7)	3,691 (82.4)	
Disabled	2,181 (16.6)	672 (15.8)	718 (16.3)	791 (17.6)	
Edentulousness, *n* (%)					0.055
Has teeth	8,757 (66.6)	2,795 (65.7)	2,915 (66.2)	3,047 (68.0)	
Edentulous	4,382 (33.4)	1,462 (34.3)	1,485 (33.8)	1,435 (32.0)	
Cognitive score, mean (SD)	0.74 (0.24)	0.71 (0.26)	0.74 (0.25)	0.78 (0.23)	<0.001
Dietary diversity					
DDS, median [IQR]	4.0 [3.0, 6.0]	2.0 [2.0, 3.0]	4.0 [4.0, 5.0]	6.0 [6.0, 7.0]	<0.001

The subgroup analyses were restricted to the BMI-complete subsample (*n* = 13,139), because BMI distribution is reported here and BMI is used as a stratification variable and as an additional covariate in sensitivity analysis (E). BMI was missing for 135 participants (1.0%) in the main analytic sample (*n* = 13,274).

ADL, activities of daily living; BMI, body mass index; CLHLS, Chinese Longitudinal Healthy Longevity Survey; DDS, dietary diversity score; IQR, interquartile range; SD, standard deviation; T1–T3, tertiles 1–3.

### 2.2 Exposure: dietary diversity score (DDS)

Dietary intake was assessed using a simplified food frequency questionnaire (FFQ) that did not record portion sizes. During face-to-face interviews, participants were asked, “How often do you currently eat this food?” The survey covered nine food groups: fresh fruits, vegetables, legumes and legume products, nuts, meat, fish, eggs, dairy products, and tea. Cereals were excluded because they are staple foods consumed almost daily in the Chinese diet, with limited inter-individual variation; cooking oils were not assessed as a distinct food-frequency item in the CLHLS and were therefore not included. The scoring rules were as follows. Fruits and vegetables were scored using four frequency categories (“almost every day”, “often”, “occasionally”, and “rarely or never”); responses of “almost every day” or “often” were assigned 1 point, with the second category for vegetables being “except in winter”, which was also considered high frequency, whereas all other responses were assigned 0 points. The remaining seven food groups were scored using five frequency categories (“almost every day,” “weekly,” “monthly,” “occasionally,” and “rarely or never”); the first two categories (consumption at least once per week) were assigned 1 point, and all other responses were assigned 0 points. The nut item referred to retrospective intake “at approximately 60 years of age”; because current nut intake was not collected in the CLHLS, this retrospective item was used as a substitute and followed the same five-category scoring rule. The DDS was calculated as the sum of scores across the nine food groups, ranging from 0 to 9. Participants with fewer than seven valid food-group responses were treated as missing, and special codes (8/9 and negative values) were also handled as missing (6). For the main analysis, DDS was categorized into tertiles: low (T1), middle (T2), and high (T3). For sensitivity analyses, it was additionally dichotomized as <5 versus ≥5. These nine food groups were measured repeatedly across all four waves, allowing lagged time-updated analyses.

### 2.3 Outcome: incident peptic ulcer disease

The outcome was incident gastric or duodenal ulcer, ascertained using item g15k1 of the CLHLS chronic disease history questionnaire (“Do you have a stomach or duodenal ulcer?”). Item g15k2 (“Was it diagnosed by a hospital?”) was used to confirm hospital diagnosis. Survey-wave-specific variables corresponded to g15k1_11/14/18. For participants who died before the next interview, ulcer status before death was reported by a family member or physician proxy (d*_gastri) and was included in the primary outcome definition. Participants with prevalent ulcer at baseline (g15k1 = 1) were excluded. Special codes were treated as missing (loss to follow-up, −9; not applicable, −7; missing, 9; “do not know,” coded as 3 in the 2011/2014 waves and 8 in the 2018 wave; and “no” for proxy death variables, coded as 0). Event time was defined as the survey wave at which an ulcer was first detected. For participants identified only through proxy reports before death, event time was assigned as the time of death (the earlier of the two times), and death was treated as a competing risk event. Analyses addressing the potential misclassification of self-reported outcomes are described in Section 2.5.

### 2.4 Covariates

The covariates included age, sex, education level, urban/rural residence, marital status, smoking, alcohol consumption, hypertension, diabetes, disability in activities of daily living (ADL), edentulism, cognitive score, and self-rated economic status. Education level was categorized according to years of schooling (f1): 0 years as illiterate, 1–6 years as primary school, 7–9 years as junior high school, and ≥10 years as senior high school or above (codes 88/99 were treated as missing). Urban/rural residence was defined by the type of residence in 2008 (residenc); urban and town areas were combined as Urban, and rural areas were classified as Rural (the CLHLS baseline survey did not collect household registration status). Marital status was dichotomized as married (f41 = 1, living with a spouse) versus other (f41 = 2–5). Smoking and alcohol consumption were categorized as never, former, or current according to current (d71/d81) and past (d72/d82) information. Cognitive score was defined as the proportion of correct answers on a 17-item cognitive test (0–1). Self-rated economic status (f34) was coded from 1 = very rich to 5 = very poor. Other chronic conditions such as coronary heart disease, stroke, and chronic lung disease were not included in the primary model. These conditions largely reflect accumulated disease burden and may themselves alter dietary intake (for example, post-stroke dysphagia); adjusting for them could therefore introduce over-adjustment or collider bias rather than reduce confounding. Because sensitivity analyses with additional adjustment for these conditions yielded materially unchanged estimates, they are reported as a sensitivity analysis (J) rather than included in the primary model. BMI may act as a mediator on the DDS–ulcer pathway; therefore, it was not included in the main model to estimate the total effect, but was additionally adjusted for in sensitivity analysis E for comparison.

### 2.5 Statistical analysis

Continuous variables are presented as means ± SD or medians (IQR), and categorical variables as *n* (%). Between-group differences were assessed using the Kruskal–Wallis and χ^2^ tests. Incidence rates were calculated as incident events divided by person-time and are expressed per 1,000 person-years.

#### 2.5.1 Main analysis

Cox proportional hazards models were fitted in three stages: Model 1, unadjusted; Model 2, adjusted for age and sex; and Model 3, additionally adjusted for education, urban/rural residence, marital status, self-rated economic status, smoking, alcohol consumption, hypertension, diabetes, ADL disability, edentulism, and cognitive score. DDS was modeled continuously (hazard ratio per 1-point increase on the 0–9 scale) and as tertiles, with the lowest tertile as the reference. Dose–response was assessed using restricted cubic splines (four knots) (13), and trend across tertiles by entering the tertile-specific median DDS (2, 4, and 6) as a continuous variable in the fully adjusted model; an integer-scoring specification (1/2/3) gave the same *p* value. Because the outcome is a time-to-event variable, the trend test was performed within the Cox model; for comparison, the Cochran–Armitage test for trend was also applied to the proportion of participants with an incident ulcer across tertiles. Since this test does not account for follow-up time or censoring, it was used only for comparison and not for primary inference (see Results and the footnote to Table 2). Effect modification was assessed using likelihood ratio tests, with subgroups defined by age group, sex, education, urban/rural residence, smoking, alcohol consumption, and BMI status. Assumptions were checked using Schoenfeld residuals, and unmeasured confounding was quantified using the E-value (14). All primary inferences were based on these standard Cox proportional hazards models with baseline DDS as the exposure; time-dependent models were used only as supplementary analyses. Although the CLHLS is a complex, multistage sampling survey, sampling weights were not applied in the primary analyses, because the objective was to examine associations within the cohort rather than to produce nationally representative estimates.

**Table 2:** Associations of dietary diversity score with incident peptic ulcer (*N* = 13,274; 636 events), CLHLS 2008–2018.

Variable	Model 1	Model 2	Model 3
DDS (per 1-point increment)			
HR (95% CI)	0.955 (0.918–0.993)	0.951 (0.914–0.989)	0.944 (0.904–0.986)
*p* value	0.022	0.013	0.009
DDS tertiles			
T1 (low)	1.00 (Ref.)	1.00 (Ref.)	1.00 (Ref.)
T2 (middle)	0.844 (0.698–1.020)	0.841 (0.696–1.017)	0.833 (0.686–1.012)
T3 (high)	0.793 (0.656–0.957)	0.779 (0.645–0.942)	0.757 (0.618–0.927)
*p* for trend	—	—	0.007

Model 1: unadjusted. Model 2: adjusted for age and sex. Model 3: fully adjusted for age, sex, education, residence, marital status, self-rated economic status, smoking, drinking, hypertension, diabetes, ADL disability, edentulousness, and cognitive score.

*p* for trend was obtained by modeling the median DDS score within each tertile (2/4/6) as a continuous variable in the fully adjusted Cox model; unlike the Cochran–Armitage test, this approach accounts for censoring and differential follow-up time. An alternative integer-scoring specification (1/2/3) yielded an identical *p* value (*p* = 0.007). For comparison, a Cochran–Armitage test of the ever/never proportion gave χ^2^ = 2.77 (p = 0.096). Incidence rates declined monotonically across tertiles (11.83, 10.13, and 9.79 per 1,000 person-years for T1, T2, and T3, respectively).

ADL, activities of daily living; CI, confidence interval; DDS, dietary diversity score; HR, hazard ratio; Ref., reference.

#### 2.5.2 Sensitivity analyses

We repeated the analysis with alternative outcome definitions, model specifications, and analytic samples: (A) a Fine–Gray competing-risk model; (B) censoring at 3 years; (B-LM6) a 6-year landmark analysis; (C) excluding baseline hypertension or diabetes; (D) binary DDS (<5 vs. ≥ 5); (E) additional adjustment for BMI; (F) directly self-reported events only; (G) hospital-confirmed events (self-report g15k1 = 1 with hospital confirmation g15k2 = 1), retaining proxy reports; (G2) hospital-confirmed self-reports only, excluding proxy reports; (H) excluding poor self-rated health at baseline; (I) multiple imputation (m = 5, pre-exclusion sample); and (J) additional adjustment for coronary heart disease, stroke, and chronic lung disease. Analyses used R 4.4.3 (survival, rms, mice), with two-sided tests and α = 0.05.

#### 2.5.3 Lagged time-updated exposure analysis

As a supplementary analysis to reduce reverse causation, a time-dependent Cox model (tmerge) divided follow-up into (0,3), (3,6), and (6,10) years. In the first specification the 2008 DDS was carried forward to the (0,3) and (3,6) intervals and the 2011 DDS assigned to the (6,10) interval, so that exposure preceded the risk period; this was used to assess robustness, not as the basis for primary inference. In a secondary wave-start specification, the 2011 and 2014 measurements were assigned to the (3,6) and (6,10) intervals, so exposure was contemporaneous with events detected in the same wave and reverse causation could not be excluded.

## 3 Results

### 3.1 Study population and baseline characteristics

A total of 16,954 participants were assessed at baseline in 2008. After excluding 391 individuals aged <65 years, 1,132 participants with prevalent peptic ulcer disease at baseline, and 2,157 individuals with missing key covariate data, 13,274 participants were included in the main analysis (Figure 1). During 60,258 person-years of follow-up (median 3.0 years; IQR 2.5–6.0), 636 incident cases of peptic ulcer were identified (4.8%), corresponding to an incidence rate of 10.6 per 1,000 person-years; 7,162 participants (54.0%) died.

**Figure 1:**
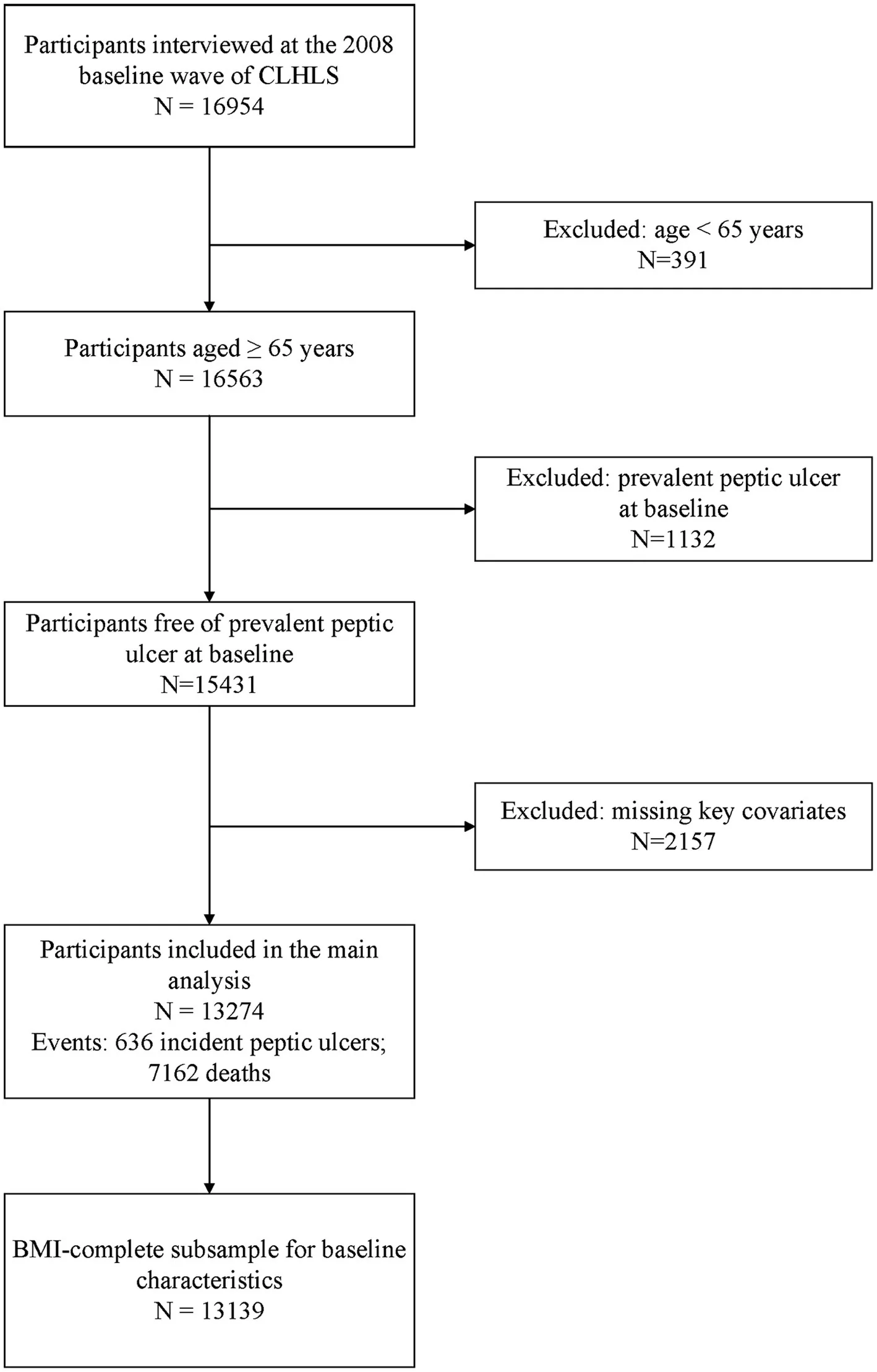
Flow diagram of participant selection. Of 16,954 participants interviewed at the 2008 baseline wave, 391 were excluded for being younger than 65 years and 1,132 for prevalent peptic ulcer at baseline, leaving 15,431 participants free of peptic ulcer. A further 2,157 were excluded because of missing data on at least one of the 13 covariates required for the fully adjusted model or on the exposure variable, leaving 13,274 participants in the main analysis (636 incident peptic ulcers; 7,162 deaths). A total of 13,139 participants with complete BMI data formed the subsample used to describe baseline characteristics. Number missing, by variable (denominator 15,431): cognitive score 1,849; hypertension 188; diabetes 121; education 41; self-rated economic status 41; edentulism 28; alcohol consumption 9; smoking 7; dietary diversity score 7. Age, sex, residence, marital status, and ADL disability had no missing values. Some participants had missing data for more than one variable, and the individual counts therefore sum to more than 2,157. BMI, body mass index; CLHLS, Chinese Longitudinal Healthy Longevity Survey.

Baseline characteristics are shown in Table 1 (BMI-complete sample, *n* = 13,139). The median age was 87 years (IQR 77–94); 7,248 participants (55.2%) were women, 7,947 (60.5%) were illiterate, 7,804 (59.4%) resided in rural areas, and 8,873 (67.5%) were classified as having “other” marital status (separated, divorced, widowed, or never married). The median DDS was 4 (IQR 3–6). After categorization into DDS tertiles, baseline characteristics differed significantly across groups (Table 1).

### 3.2 Association between DDS and incident ulcer

Among the 636 incident ulcer cases, DDS was inversely associated with ulcer incidence (Table 2). In the continuous analysis, each 1-point increase in DDS was associated with a lower hazard of ulcer in Model 1 (unadjusted; HR = 0.955, 95% CI 0.918–0.993, *p* = 0.022), Model 2 (adjusted for age and sex; HR = 0.951, 95% CI 0.914–0.989, *p* = 0.013), and Model 3 (fully adjusted; HR = 0.944, 95% CI 0.904–0.986, *p* = 0.009), corresponding to an approximately 5.6% reduction in ulcer risk per 1-point increase in DDS. In the tertile analysis, compared with T1, the risk was significantly lower in T3 (Model 3: HR = 0.757, 95% CI 0.618–0.927, *p* = 0.007), corresponding to an approximately 24% lower risk, whereas T2 did not differ significantly from T1 (HR = 0.833, 95% CI 0.686–1.012, *p* = 0.065). A graded decreasing trend in risk was observed from T1 to T3 (*p* for trend = 0.007). Across the three models, the HRs for T3 were 0.793 (95% CI 0.656–0.957), 0.779 (95% CI 0.645–0.942), and 0.757 (95% CI 0.618–0.927), indicating minimal change after adjustment. Schoenfeld residual tests did not indicate violation of the proportional hazards assumption (DDS, *p* = 0.211; global test, *p* = 0.845).

Because the trend result may depend on the test used, both approaches were applied (Table 2, footnote). With the median DDS within each tertile (2, 4, 6) entered as a continuous term in the fully adjusted Cox model, the hazard ratio per one-tertile increase was 0.932 (95% CI 0.886–0.981; *p* = 0.007). A Cochran–Armitage test of the ever/never proportion, which takes no account of person-time, gave χ^2^ = 2.77 (*p* = 0.096). The two tests agreed in direction, both indicating the highest risk in the lowest tertile, but only the Cox-based test reached statistical significance. The proportion ever affected was 5.31%, 4.53%, and 4.55% across tertiles, whereas the incidence rate declined monotonically (11.83, 10.13, and 9.79 per 1,000 person-years); mortality differed appreciably across the three groups (58.4%, 55.6%, and 48.1%).

### 3.3 Dose–response relationship

In the restricted cubic spline analysis, using DDS = 4 as the reference, ulcer risk decreased monotonically with increasing DDS. The HR declined from 1.42 (95% CI 0.92–2.21) at DDS = 0 to 0.88 (95% CI 0.58–1.33) at DDS = 9 (with four knots placed at DDS = 1, 4, 5, and 8). The hazard ratios were 1.18 (95% CI 1.02–1.37) at DDS = 2 and 1.08 (95% CI 1.01–1.16) at DDS = 3, indicating a modestly higher risk below the reference value; at DDS = 0, 1, and 5–9, the confidence intervals included 1. No evidence of non-linearity was observed (*p* for non-linearity = 0.655; Figure 2), consistent with the linear estimate in Section 3.2 showing an HR of 0.944 (95% CI 0.904–0.986) per 1-point increase in DDS. Kaplan–Meier curves stratified by DDS tertiles separated early in follow-up (number at risk at baseline: 4,291, 4,455, and 4,528 in T1, T2, and T3, respectively). Throughout follow-up, cumulative ulcer incidence remained highest in T1 and lowest in T3 (log-rank *p* = 0.041; Figure 3A, B).

**Figure 2:**
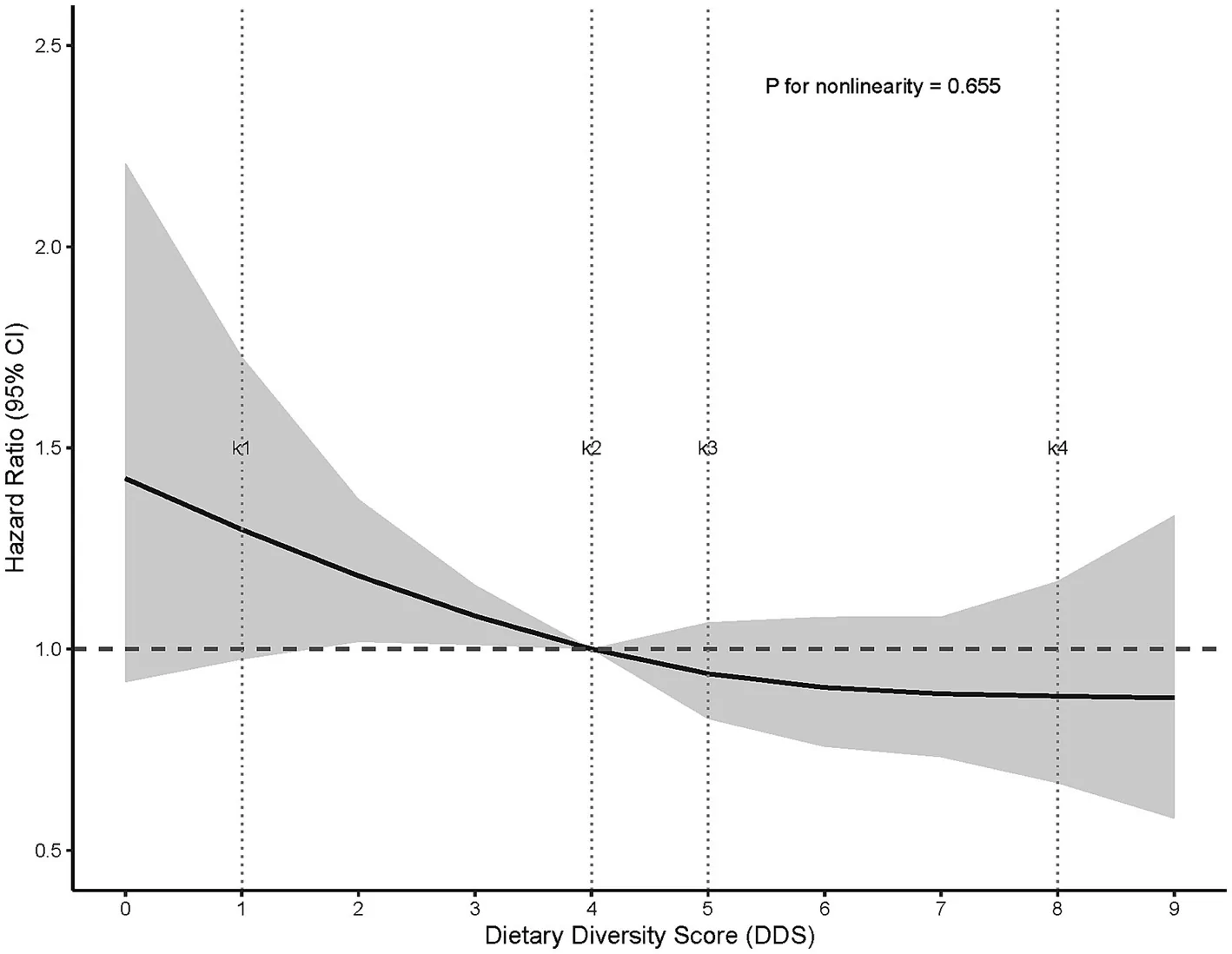
Dose–response relationship between DDS and incident peptic ulcer. Dose–response relationship between the dietary diversity score (continuous) and the risk of incident peptic ulcer, estimated by restricted cubic splines with 4 knots (dashed vertical lines) within a Cox proportional hazards model adjusted for the full covariate set as in Model 3 of Table 2 (BMI not included). The solid curve represents the hazard ratio and the shaded area the 95% confidence interval, both referenced to DDS = 4 (the median); the reference is shown as a horizontal line at HR = 1. CI, confidence interval; DDS, dietary diversity score; HR, hazard ratio.

**Figure 3:**
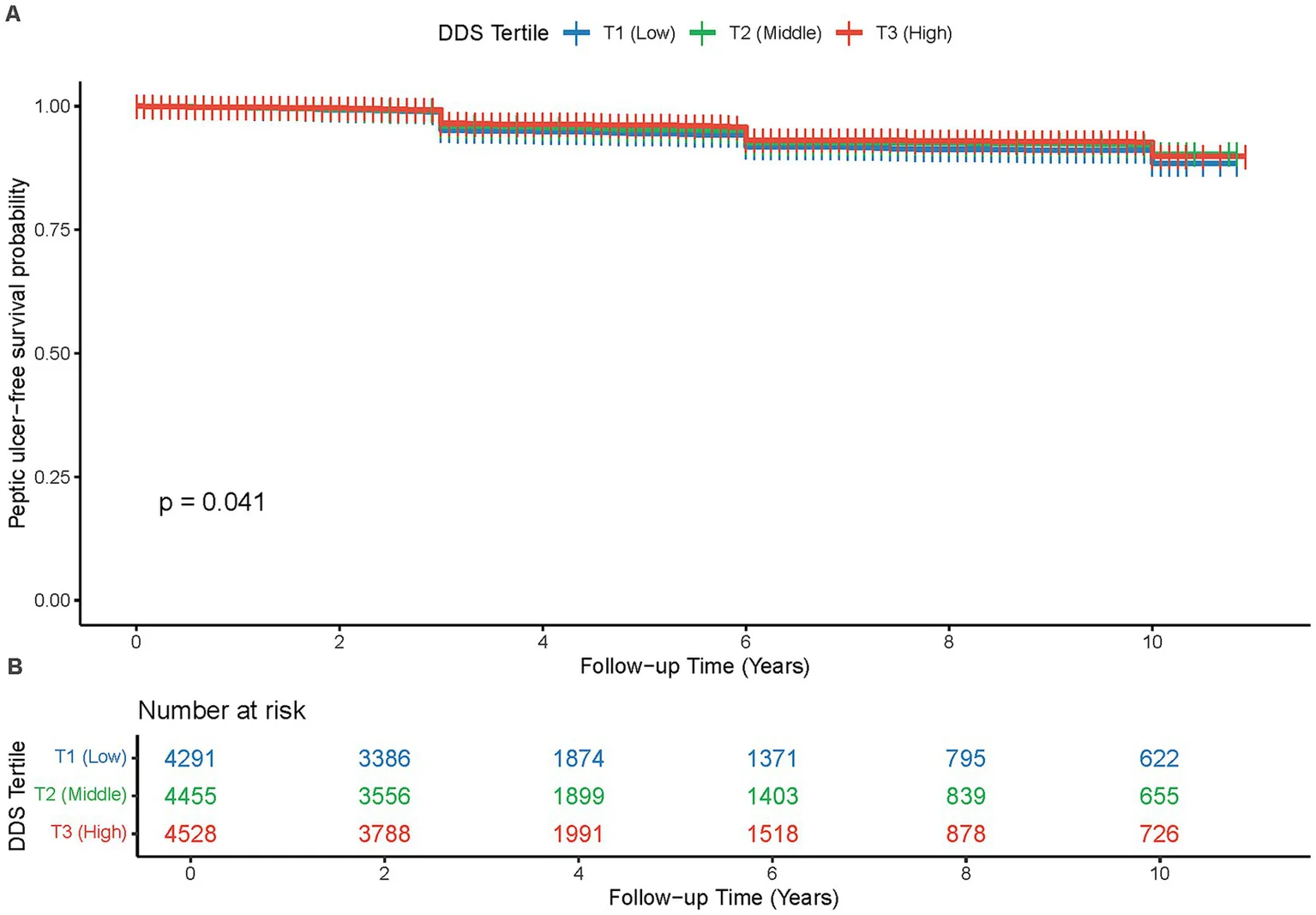
Kaplan–Meier curves by DDS tertile **(A)** Kaplan–Meier curves for peptic ulcer-free survival by tertiles of the dietary diversity score (T1, lowest; T2, middle; T3, highest); the log-rank *p* value is shown. **(B)** Number of participants at risk at each follow-up time point, corresponding to the curves in **(A)**. Curves were based on the main analytic sample (*n* = 13,274); Table 1 used the BMI-complete sample (*n* = 13,139). DDS, dietary diversity score; T1–T3, tertiles 1–3.

### 3.4 Subgroup analyses

Subgroup and interaction analyses were conducted in the BMI-complete sample (*n* = 13,139). Within each stratum, models were adjusted for age (continuous), sex, education, urban/rural residence, marital status, smoking, alcohol consumption, hypertension, diabetes, ADL disability, and BMI, with the stratifying variable itself excluded (for the age strata, the continuous age term was also excluded); interaction tests were performed using likelihood ratio tests for nested models (Figure 4). The inverse association between DDS and ulcer risk was directionally consistent across most prespecified subgroups. Except for ADL status, no significant effect modification was observed by age (*p* = 0.220), sex (*p* = 0.896), urban/rural residence (*p* = 0.796), education (*p* = 0.794), smoking (*p* = 0.645), alcohol consumption (*p* = 0.121), or BMI (*p* = 0.411). Effect modification by ADL status was significant (*p* = 0.004): among participants without ADL disability, each 1-point increase in DDS was associated with a lower risk of ulcer (HR = 0.95, 95% CI 0.91–0.99, *p* = 0.019), whereas no significant association was observed among participants with ADL disability (HR = 1.02, 95% CI 0.91–1.15, *p* = 0.702). The association was also statistically significant among participants aged <80 years (HR = 0.94, 95% CI 0.88–1.00, *p* = 0.045), men (HR = 0.94, 95% CI 0.88–1.00, *p* = 0.036), and never-smokers (HR = 0.94, 95% CI 0.89–0.99, *p* = 0.030).

**Figure 4:**
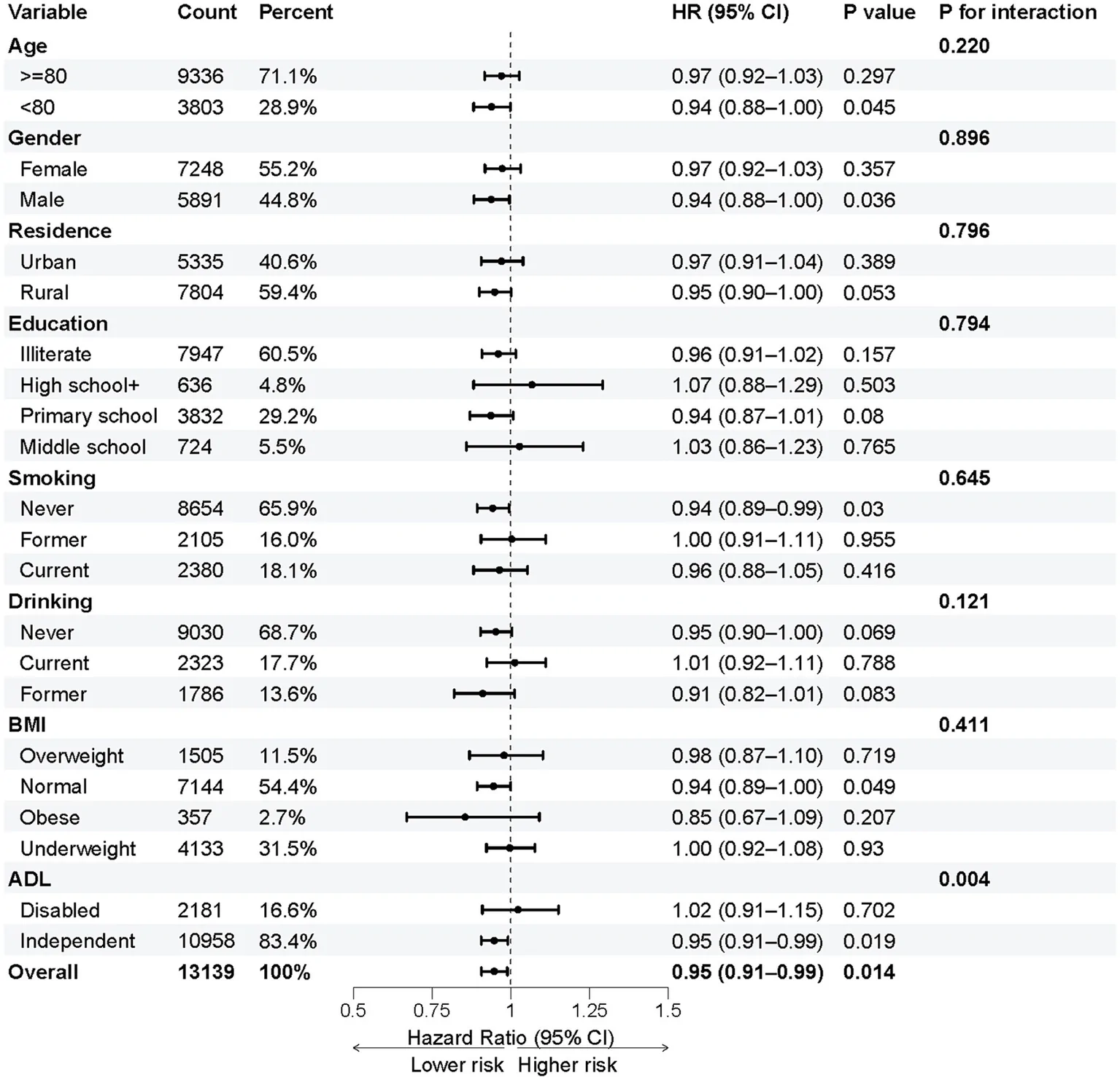
Subgroup analyses of the association between the dietary diversity score (per 1-point increment) and incident peptic ulcer. Models were adjusted for age (continuous), sex, education, urban/rural residence, marital status, smoking, alcohol consumption, hypertension, diabetes, ADL disability, and BMI; the stratifying variable was excluded from its respective subgroup model. *p* for interaction was estimated using likelihood ratio tests. Analyses were based on the BMI-complete subsample (*n* = 13,139); therefore, the overall estimate (HR = 0.95) differs slightly from the main analysis (HR = 0.944, 95% CI 0.904–0.986, *n* = 13,274), which did not adjust for BMI. ADL, activities of daily living; BMI, body mass index; CI, confidence interval; DDS, dietary diversity score; HR, hazard ratio.

### 3.5 Sensitivity analyses

The results of the sensitivity analyses are presented in Table 3. In the Fine–Gray model treating death as a competing risk, the association remained significant (HR = 0.955, 95% CI 0.914–0.997, *p* = 0.035). Similar effect estimates were observed after using hospital-confirmed diagnoses (G: HR = 0.957, 95% CI 0.915–1.001, *p* = 0.058; G2: HR = 0.945, 95% CI 0.895–0.998, *p* = 0.041), restricting the outcome to self-reported events only (F: HR = 0.931, 95% CI 0.885–0.979, *p* = 0.005), excluding participants with poor self-rated health at baseline (H: HR = 0.938, 95% CI 0.895–0.983, *p* = 0.007), and applying multiple imputation (I: HR = 0.946, 95% CI 0.908–0.985, *p* = 0.008). The association also persisted after additional adjustment for BMI (E: HR = 0.950, 95% CI 0.910–0.993, *p* = 0.022). When DDS was dichotomized as <5 versus ≥5, the high-DDS group showed a lower risk, although this was not statistically significant (D: HR = 0.863, 95% CI 0.732–1.019, *p* = 0.082). After excluding participants with baseline hypertension or diabetes, the association was slightly attenuated (C: HR = 0.962, 95% CI 0.916–1.010, *p* = 0.122).

**Table 3:** Sensitivity analyses for the association between dietary diversity score and incident peptic ulcer (*N* = 13,274; 636 events).

Analysis	Events	HR (95% CI)	*p*	*E*-value
Main analysis (cause-specific Cox)	636	0.944 (0.904–0.986)	0.009	1.31
(A) Fine-Gray competing risk model	636	0.955 (0.914–0.997)	0.035	1.27
(B) Censor early events at 3 years	229	0.996 (0.928–1.070)	0.920	1.06
(C) Excluding baseline hypertension/diabetes	488	0.962 (0.916–1.010)	0.122	1.24
(D) DDS binary (high vs. low)	636	0.863 (0.732–1.019)	0.082	1.59
(E) Additional adjustment for BMI	—	0.950 (0.910–0.993)	0.022	1.29
(F) Self-reported ulcer only (no proxy)	—	0.931 (0.885–0.979)	0.005	1.36
(G) Hospital-confirmed ulcer (g15k2 = 1, proxy retained)	575	0.957 (0.915–1.001)	0.058	1.26
(G2) Hospital-confirmed ulcer, self-report only	402	0.945 (0.895–0.998)	0.041	1.31
(H) Excluding baseline poor self-rated health	539	0.938 (0.895–0.983)	0.007	1.33
(I) Multiple imputation (m = 5, pre-CC sample)	—	0.946 (0.908–0.985)	0.008	1.30
(P4) Lagged time-updated DDS, baseline-persistent mapping	525	0.944 (0.900–0.990)	0.017	1.31
(P4-WS) Lagged time-updated DDS, wave-start mapping	525	0.914 (0.871–0.959)	<0.001	1.42
(B-LM6) Landmark analysis at 6 years	68	0.988 (0.865–1.128)	0.856	1.12
(J) Additionally adjusted for coronary heart disease, stroke, and chronic lung disease	635	0.943 (0.903–0.985)	0.008	1.31

All models were adjusted for the full set of covariates as in Model 3 of Table 2, unless stated otherwise. HRs are for a 1-point increment in DDS.

Events: number of incident peptic ulcer events in the corresponding analysis sample; (E) was based on the BMI-complete sample (*n* = 13,139); (F) counted self-reported events only; (I) was based on multiple imputation without a single event count.

(P4) used the baseline-persistent mapping, in which exposure was measured strictly before each risk period (presented for robustness). (P4-WS) used the wave-start (proximal) mapping, which includes same-wave exposure measurement for the (3,6) interval and is presented for robustness only. (P4) and (P4-WS) required non-missing exposure measurements at both the 2008 and 2011 waves and were therefore restricted to 7,062 participants (525 events); most of the participants not included had died before the 2011 wave, which explains why the event count is lower than in the main analysis. In (P4-WS), the 2014 exposure was carried forward from 2011 for 2,718 participants (38.5%) without a 2014 measurement; (J) additionally adjusted for coronary heart disease (g15c1), stroke (g15d1), and chronic lung disease (g15e1). Of the 13,274 participants in the main analysis, 64 had missing data on at least one of these three items (coronary heart disease 23; stroke 30; chronic lung disease 24; 13 participants had more than one item missing), leaving 13,210 participants and 635 events. The estimate was materially unchanged, indicating that the results were not sensitive to the comorbidity covariate set.

E-values denote the minimum strength of association an unmeasured confounder would need to have with both exposure and outcome to fully explain the observed association, based on the HR≈RR approximation. E-values in this table are based on point estimates; the more conservative E-values based on the confidence limit closest to the null are reported in the text.

BMI, body mass index; CI, confidence interval; DDS, dietary diversity score; HR, hazard ratio; MI, multiple imputation; OR, odds ratio; P4, lagged time-updated exposure (baseline-persistent mapping); P4-WS, lagged time-updated exposure (wave-start mapping); RR, risk ratio.

When early events were censored at 3 years (B: HR = 0.996, 95% CI 0.928–1.070, *p* = 0.920) and in the 6-year landmark analysis (B-LM6: HR = 0.988, 95% CI 0.865–1.128, *p* = 0.856), the association was attenuated to null, suggesting that the predictive value of DDS was concentrated in early follow-up. In the time-updated lagged analyses, the baseline exposure carried-forward approach (P4) yielded an HR of 0.944 (95% CI 0.900–0.990, *p* = 0.017), whereas the wave-start mapping approach (P4-WS) yielded an HR of 0.914 (95% CI 0.871–0.959, *p* < 0.001); the association persisted under both exposure-mapping strategies, in which exposure was measured strictly before the event or contemporaneously with it. The E-value for the main analysis was 1.31 based on the point estimate and 1.13 based on the confidence limit closest to 1. Additional adjustment for coronary heart disease, stroke, and chronic lung disease (J) left the estimate essentially unchanged (HR = 0.943, 95% CI 0.903–0.985, *p* = 0.008).

## 4 Discussion

This study included 13,274 Chinese older adults. Baseline DDS was inversely associated with the risk of incident peptic ulcer: each 1-point increase in DDS was associated with an approximately 5.6% lower risk (HR = 0.944, 95% CI 0.904–0.986). Compared with the lowest tertile, the highest tertile had an approximately 24% lower risk (HR = 0.757, 95% CI 0.618–0.927; trend test *p* = 0.007). A Cochran–Armitage test and a Cox-based trend test do not estimate the same quantity. The first compares the proportion of participants ever affected across ordered categories, assuming a fixed period of observation; the second compares the hazard, the event rate per unit of time at risk. Mortality differed appreciably across tertiles (58.4%, 55.6%, and 48.1%), so the crude proportion combines the rate of ulcer occurrence with the time each group was observed. Deaths were most frequent in the lowest tertile, whose observation was therefore truncated and whose crude proportion was reduced rather than raised. The two tests agreed in direction and differed only in significance; the Cochran–Armitage result reflects the information lost when person-time and the 7,162 deaths are disregarded. For this reason, the primary inference rests on the Cox model. Dose–response analyses did not show evidence of deviation from linearity (non-linearity test *p* = 0.655). The association was directionally consistent across multiple analytic specifications, including competing-risk models for death (Fine–Gray HR = 0.955), hospital-confirmed diagnoses (G2 HR = 0.945), self-reported cases only (HR = 0.931), exclusion of baseline subclinical conditions (HR = 0.938), and multiple imputation analyses (HR = 0.946). The association also persisted in lag analyses in which exposure assessment was strictly separated from the risk period (P4: HR = 0.944, *p* = 0.017). However, both 3-year censoring and 6-year landmark analyses indicated that the effect was concentrated primarily in the early follow-up period. In addition, the interaction with ADL status was significant (*p* = 0.004), and the protective association was observed only among functionally independent participants. These findings suggest that greater dietary diversity is associated with a lower risk of ulcer incidence in older adults; however, the association appears to be largely confined to early follow-up and may partly reflect overall health status. The long-term effect therefore remains to be confirmed.

Previous dietary studies on ulcer risk have largely focused on single nutrients or specific dietary patterns. Aldoori et al. reported an inverse association between dietary fiber intake and duodenal ulcer risk among American men (8). Kurata et al. suggested that dietary factors were associated with ulcer risk in Seventh-day Adventists (9). More recently, two studies of overall dietary patterns provided complementary evidence: in a Korean cohort, a higher phytochemical index was associated with a lower risk of gastritis/gastric ulcer (15), whereas in the Spanish SUN cohort, higher ultra-processed food intake was associated with an increased risk of ulcer incidence (16). In contrast to these studies, we assessed diet quality from the perspective of “food-group diversity,” which differs conceptually from the exposures used in prior work. Moreover, our study population consisted of very old adults (median age, 87 years), among whom age-related changes such as gastric mucosal atrophy and reduced blood supply are common (17); both ulcer occurrence and competition from mortality therefore differ from those in younger populations. To our knowledge, this is among the first studies to examine the association between dietary diversity and incident ulcer in a longevity population. Prior DDS studies based on the CLHLS have focused on outcomes such as mortality, frailty, sarcopenia, and ADL disability (6, 10–12). The present study adds digestive outcomes to this body of evidence.

The protective association of DDS was observed only among participants who were independent in activities of daily living (ADL) (HR = 0.95, 95% CI 0.91–0.99) and was absent among those with ADL disability (HR = 1.02, 95% CI 0.91–1.15), with a significant interaction test (*p* = 0.004). This may be because older adults with disability often experience impaired oral function and reduced appetite; although their food groups may appear diverse, actual intake and absorption may be insufficient, making it difficult for dietary diversity to translate into mucosal protection (12, 18, 19). Consistently, we also found an association between edentulism and DDS score, which supports this interpretation. Ulcer healing depends on mucosal epithelial proliferation and adequate blood supply (20), whereas individuals with disability have diminished overall reparative reserve (21), which may not be readily compensated by dietary substrates alone (22). Because participants with disability had poorer baseline health, a low DDS is more likely to reflect disease-related dietary changes; reverse causation may therefore play a larger role in this subgroup and could attenuate the prospective association. In addition, participants with disability have a higher risk of death (23), which reduces the likelihood that ulcer events are detected before death. Although competing-risk methods were applied in the main analysis, residual influence may still persist within the subgroup. These observations are consistent with the association being partly mediated by overall health status and physical function, rather than reflecting a purely direct dietary–mucosal effect.

Older adults with greater dietary diversity tend to have a more balanced nutrient intake (7), which may protect the gastric mucosa through several pathways: protein, zinc, and vitamin A help maintain mucosal epithelial renewal and mucus secretion (24–26); antioxidant compounds such as polyphenols and carotenoids in plant foods scavenge reactive oxygen species and mitigate mucosal oxidative damage in the setting of *H. pylori* infection and NSAID exposure (27, 28); and short-chain fatty acids produced by the fermentation of dietary fiber exert anti-inflammatory effects (29). On the other hand, older adults with higher DDS also tended to have higher educational attainment and cognitive function, but they also had a higher prevalence of current smoking and alcohol consumption (Table 1). Dietary diversity may therefore be influenced by education and cognitive status, and the observed association may reflect a true dietary–mucosal effect as well as unmeasured factors related to health awareness.

The association disappeared in the 3-year censoring analysis (HR = 0.996) and the 6-year landmark analysis (HR = 0.988), whereas it became stronger in the wave-based alignment of updated exposure (HR = 0.914, *p* < 0.001). Three explanations are plausible. First, reverse causation cannot be excluded: declining health or early, undiagnosed ulcer may have led older adults to reduce or alter their dietary intake before baseline, and such disease-driven dietary changes would be concentrated in the early follow-up period and largely exhausted within 3 years, which is consistent with the null association after 3-year censoring. Second, baseline DDS may no longer represent current diet over time, and the association re-emerged after wave-based updating of exposure (HR = 0.914, *p* < 0.001), suggesting that the dietary effect may be better characterized as a “recent exposure–recent protection” relationship rather than a sustained cumulative effect. Third, the 6-year landmark analysis included only 68 events, resulting in a wide confidence interval (0.865–1.128); a true effect therefore cannot be excluded, even if it was not detected. Observational data cannot distinguish among these mechanisms, and the long-term causal effect will require confirmation in intervention studies.

This study was based on a large longevity cohort and used competing-risk models, hospital-confirmed diagnoses, and lagged time-updated analyses; however, several limitations should be noted. First, the CLHLS did not collect data on NSAID use or *H. pylori* infection, both of which are strongly associated with ulcer risk (5, 30, 31). The E-value for the main analysis was 1.31 based on the point estimate and 1.13 based on the confidence limit closest to 1, indicating that residual confounding by unmeasured factors cannot be excluded. However, evidence linking NSAID use or *H. pylori* infection to dietary diversity is limited and inconsistent (32, 33), and the direction of any such confounding is uncertain: neither factor is clearly associated with higher dietary diversity, and confounding could plausibly bias the estimate toward the null as well as away from it. Residual confounding is therefore unlikely to fully explain the observed association, although this possibility cannot be ruled out. Confirmation in studies with detailed medication and infection data is warranted. Second, ulcer cases were identified primarily by self-report, and for decedents the information was provided by family proxies without confirmation against hospital records; ulcers that occurred and healed between waves, or that remained undiagnosed at death, may also have been missed. Nevertheless, the association remained materially unchanged when outcomes were restricted to hospital-confirmed cases with proxy events retained (G) or to hospital-confirmed self-reports only (G2), suggesting that the impact of outcome misclassification was limited. Third, event times were approximated by survey waves, representing a simplified approach to interval censoring that is standard in equally spaced follow-up cohorts such as the CLHLS. Fourth, the cohort consisted of very old survivors, which may introduce survivor selection bias. Fifth, the nut item assessed retrospective frequency “at approximately age 60,” which may be subject to recall bias, and the composite DDS is therefore not strictly contemporaneous, because this item reflects intake at age ~60 whereas the other eight items reflect current intake. The CLHLS did not collect information on current nut intake, and this retrospective item is a commonly used substitute. Sixth, the simplified FFQ did not include quantitative information, precluding direct adjustment for total energy intake; instead, energy intake was controlled indirectly through covariates. Seventh, sampling weights were not applied. Given the CLHLS sampling design, which oversamples the oldest old, our results should be interpreted as reflecting associations within the study cohort rather than being fully representative of the entire older Chinese population; in particular, the reported incidence rate is an unweighted within-cohort rate rather than a national estimate.

## 5 Conclusion

This study indicates that greater dietary diversity is associated with a lower risk of incident peptic ulcer among longevity older adults. The association was mainly observed during early follow-up and was evident only among functionally independent participants, suggesting that the protective effect of dietary diversity may be partly mediated by overall health status. The DDS is based on only nine food-frequency items and is easy to administer, which may facilitate its use in dietary assessment in clinical settings. However, its long-term causal effect and potential value for intervention warrant confirmation in randomized studies.

## Data Availability

Publicly available datasets were analyzed in this study. This data can be found at: https://opendata.pku.edu.cn/dataset.xhtml?persistentId=doi:10.18170/DVN/WBO7LK&version=2.0.

## References

[1] Lanas A Chan FKL. Peptic ulcer disease. Lancet. (2017) 390:613–24. doi: 10.1016/S0140-6736(16)32404-7 28242110

[2] Yang H Zhang M Li H Huang Z Sun Y Li W . Prevalence of common upper gastrointestinal diseases in Chinese adults aged 18-64 years. Sci Bull. (2024) 69:3889–98. doi: 10.1016/j.scib.2024.07.048, 39562185

[3] Costable NJ Greenwald DA. Upper gastrointestinal bleeding. Clin Geriatr Med. (2021) 37:155–72. doi: 10.1016/j.cger.2020.09.001 33213769

[4] Xie L Liu G-W Liu Y-N Li P-Y Hu X-N He X-Y . Prevalence of *helicobacter pylori* infection in China from 2014-2023: a systematic review and meta-analysis. World J Gastroenterol. (2024) 30:4636–56. doi: 10.3748/wjg.v30.i43.4636, 39575409 PMC11572641

[5] Ko KA Lee D-K. Nonsteroidal anti-inflammatory drug-induced peptic ulcer disease. Korean J Helicobacter Up Gastrointest Res. (2025) 25:34–41. doi: 10.7704/kjhugr.2025.0004, 40550526 PMC12173566

[6] Li X Wang F Guo R Liu Y Wu Z Han Y . Muscle mass mediates the association between dietary diversity and mortality among the older adults: a prospective cohort study. Clin Nutr. (2025) 47:21–7. doi: 10.1016/j.clnu.2025.02.004, 39978231

[7] Kong W Jiang T Ning Y Guo Y Liu H Lyu X . Dietary diversity, diet quality, and oxidative stress in older adults. Geriatr Nurs. (2022) 48:158–63. doi: 10.1016/j.gerinurse.2022.09.013, 36219935

[8] Aldoori WH Giovannucci EL Stampfer MJ Rimm EB Wing AL Willett WC. Prospective study of diet and the risk of duodenal ulcer in men. Am J Epidemiol. (1997) 145:42–50. doi: 10.1093/oxfordjournals.aje.a009030, 8982021

[9] Kurata JH Nogawa AN Abbey DE Petersen F. A prospective study of risk for peptic ulcer disease in seventh-day adventists. Gastroenterology. (1992) 102:902–9. doi: 10.1016/0016-5085(92)90176-Y, 1537526

[10] Duan Y Qi Q Cui Y Yang L Zhang M Liu H. Effects of dietary diversity on frailty in Chinese older adults: a 3-year cohort study. BMC Geriatr. (2023) 23:141. doi: 10.1186/s12877-023-03875-5, 36918767 PMC10012609

[11] Du Q Lu Y Hu F Feng X Zhang Y Li S . Dietary diversity and possible sarcopenia among older people in China: a nationwide population-based study. Front Nutr. (2023) 10:1218453. doi: 10.3389/fnut.2023.1218453, 37457980 PMC10348914

[12] Huang L Liu Z Wang W Zhao R Sun P Xu Y . Association of dietary diversity with disability in activities of daily living among older Chinese people: a longitudinal study. BMC Geriatr. (2025) 25:770. doi: 10.1186/s12877-025-06397-4, 41083941 PMC12519726

[13] Desquilbet L Mariotti F. Dose-response analyses using restricted cubic spline functions in public health research. Stat Med. (2010) 29:1037–57. doi: 10.1002/sim.3841, 20087875

[14] VanderWeele TJ Ding P. Sensitivity analysis in observational research: introducing the E-value. Ann Intern Med. (2017) 167:268–74. doi: 10.7326/M16-2607, 28693043

[15] Park Y Park K. Phytochemical index and the risk of gastritis/gastric ulcer among korean adults: a prospective cohort study. Nutrients. (2024) 16:2514. doi: 10.3390/nu16152514, 39125393 PMC11314585

[16] Leone A De la Fuente-Arrillaga C Mas MV Sayon-Orea C Menichetti F Martínez-Gonzalez MA . Association between the consumption of ultra-processed foods and the incidence of peptic ulcer disease in the SUN project: a Spanish prospective cohort study. Eur J Nutr. (2024) 63:2367–78. doi: 10.1007/s00394-024-03439-2, 38809325 PMC11377682

[17] Salles N. Is stomach spontaneously ageing? Pathophysiology of the ageing stomach. Best Pract Res Clin Gastroenterol. (2009) 23:805–19. doi: 10.1016/j.bpg.2009.09.002, 19942159

[18] Wang Y Sun X. The effect of tooth loss on depression and anxiety among older adults in China: the mediating role of dietary diversity. Nutrients. (2026) 18:893. doi: 10.3390/nu18060893, 41901068 PMC13028850

[19] Fielding RA Landi F Smoyer KE Tarasenko L Groarke J. Association of anorexia/appetite loss with malnutrition and mortality in older populations: a systematic literature review. J Cachexia Sarcopenia Muscle. (2023) 14:706–29. doi: 10.1002/jcsm.13186, 36807868 PMC10067499

[20] Hagen SJ. Gastroduodenal injury and repair mechanisms. Curr Opin Gastroenterol. (2024) 40:477–83. doi: 10.1097/MOG.0000000000001049, 38935320

[21] de Sire A Ferrillo M Lippi L Agostini F de Sire R Ferrara PE . Sarcopenic dysphagia, malnutrition, and oral frailty in elderly: a comprehensive review. Nutrients. (2022) 14:982. doi: 10.3390/nu14050982, 35267957 PMC8912303

[22] Langer G Wan CS Fink A Schwingshackl L Schoberer D. Nutritional interventions for preventing and treating pressure ulcers. Cochrane Database Syst Rev. (2024) 2:CD003216. doi: 10.1002/14651858.CD003216.pub3, 38345088 PMC10860148

[23] Liu L Zheng Y Tian J Li L Ruan H Jia S . Disparities in overall survival by varying duration of disability in activities of daily living in older people: a population-based cohort from Chinese longitudinal healthy longevity survey (CLHLS). J Nutr Health Aging. (2024) 28:100022. doi: 10.1016/j.jnha.2023.100022, 38267151 PMC12877755

[24] Efthymakis K Neri M. The role of zinc L-carnosine in the prevention and treatment of gastrointestinal mucosal disease in humans: a review. Clin Res Hepatol Gastroenterol. (2022) 46:101954. doi: 10.1016/j.clinre.2022.101954, 35659631

[25] Vomero ND Colpo E. Nutritional care in peptic ulcer. Arq Bras Cir Dig. (2014) 27:298–302. doi: 10.1590/S0102-67202014000400017, 25626944 PMC4743227

[26] Beaumont M Blachier F. Amino acids in intestinal physiology and health. Adv Exp Med Biol. (2020) 1265:1–20. doi: 10.1007/978-3-030-45328-2_1, 32761567

[27] Kim H. Oxidative stress in *Helicobacter pylori*-induced gastric cell injury. Inflammopharmacology. (2005) 13:63–74. doi: 10.1163/156856005774423962 16259728

[28] Yan S Bao S Chen T Chen J Zhang J Hu X . Cinnamaldehyde alleviates aspirin-induced gastric mucosal injury by regulating pi3k/akt pathway-mediated apoptosis, autophagy and ferroptosis. Phytomedicine. (2024) 132:155791. doi: 10.1016/j.phymed.2024.155791, 38901284

[29] Zhang D Jian Y-P Zhang Y-N Li Y Gu L-T Sun H-H . Short-chain fatty acids in diseases. Cell Commun Signal. (2023) 21:212. doi: 10.1186/s12964-023-01219-9, 37596634 PMC10436623

[30] Prescrire International. *Helicobacter pylori* and gastric or duodenal ulcer. Prescrire Int. (2016) 25:18–23. 26942258

[31] Leodolter A Kulig M Brasch H Meyer-Sabellek W Willich SN Malfertheiner P. A meta-analysis comparing eradication, healing and relapse rates in patients with *helicobacter pylori*-associated gastric or duodenal ulcer. Aliment Pharmacol Ther. (2001) 15:1949–58. doi: 10.1046/j.1365-2036.2001.01109.x, 11736726

[32] Rocco A Nardone G. Diet, H pylori infection and gastric cancer: evidence and controversies. World J Gastroenterol. (2007) 13:2901–12. doi: 10.3748/wjg.v13.i21.2901, 17589938 PMC4171140

[33] Kasum VU Hayati F Syed Abdul Rahim SS Nik Lah NAS Tung SEH. Association between dietary pattern and *Helicobacter pylori* infection at Queen Elizabeth Hospital, Kota Kinabalu: a case-control study. Asian J Surg. (2024) 47:3852–7. doi: 10.1016/j.asjsur.2024.03.177 38604869

